# Neuromuscular electrical stimulation to combat cognitive aging in people with spinal cord injury: protocol for a single case experimental design study

**DOI:** 10.1186/s12883-024-03699-9

**Published:** 2024-06-11

**Authors:** Wouter A. J. Vints, Oron Levin, Martijn van Griensven, Johan W. S. Vlaeyen, Nerijus Masiulis, Jeanine Verbunt, Charlotte C. M. van Laake-Geelen

**Affiliations:** 1 Department of Health Promotion and Rehabilitation, Lithuanian Sports University, Sporto Str. 6, Kaunas, LT-44221 Lithuania; 2 Department of Rehabilitation Medicine Research School CAPHRI, Maastricht University, P.O. Box 616, Maastricht, 6200 MD The Netherlands; 3 Centre of Expertise in Rehabilitation and Audiology, Adelante Zorggroep, P.O. Box 88, Hoensbroek, 6430 AB The Netherlands; 4 Movement Control & Neuroplasticity Research Group, Group Biomedical Sciences, KU Leuven, Tervuursevest 101, Heverlee, 3001 Belgium; 5 Department of Cell Biology-Inspired Tissue Engineering, MERLN Institute for Technology-Inspired Regenerative Medicine, Maastricht University, P.O. Box 616, Maastricht, 6200 MD The Netherlands; 6 Experimental Health Psychology, Faculty of Psychology and Neuroscience, Maastricht University, Maastricht, Netherlands; 7 Health Psychology Research Group, Faculty of Psychology and Educational Sciences, KU Leuven, Tiensestraat 102, Louvain, 3000 Belgium; 8 Department of Rehabilitation, Physical and Sports Medicine, Faculty of Medicine, Institute of Health Science, Vilnius University, M. K. Čiurlionio Str. 21, Vilnius, 03101 Lithuania

**Keywords:** Spinal cord injury, Myokine, Neuromuscular electrical stimulation, Cognition, Neuroplasticity, Brain-derived neurotrophic factor, Single case experimental design, Chronic rehabilitation care

## Abstract

**Introduction:**

Individuals with spinal cord injury (SCI) can experience accelerated cognitive aging. Myokines (factors released from muscle cells during contractions), such as brain-derived neurotrophic factor (BDNF), are thought to have beneficial effects on cognition. Neuromuscular electrical stimulation (NMES) was shown to elicit a large release of myokines. However, the effects of NMES on cognitive function have not been studied.

**Objective:**

To present the study protocol for a clinical trial evaluating the effects of NMES aimed at improving cognition and BDNF.

**Methods:**

A replicated randomized three-phases single-case experimental design (SCED) with sequential multiple baseline time series and a single-armed prospective trial will be conducted with 15 adults with chronic SCI (> 12 months after injury) above L1 neurological level undergoing 30-min quadriceps NMES, 3 days per week for 12 weeks.

**Main study endpoints:**

Primary endpoint is cognitive performance (assessed by a smartphone test) conducted three times per week during the baseline phase with random duration of 3 to 8 weeks, the intervention phase of 12 weeks, and the follow-up phase of 3 weeks after a no measurement rest period of 12 weeks. Secondary endpoints are changes in BDNF levels and cognitive performance measured before the baseline period, before and after intervention and after a 12 weeks follow-up.

**Conclusion:**

This will be the first study investigating the effects of 12 weeks NMES on both cognition and BDNF levels in individuals with SCI. The SCED results provide information on individual treatment effect courses which may direct future research.

**Trial registration:**

ClinicalTrials.gov (NCT05822297, 12/01/2023).

**Supplementary Information:**

The online version contains supplementary material available at 10.1186/s12883-024-03699-9.

## Introduction

Cognitive problems in persons with spinal cord injury (SCI) have long been underrecognized and were usually explained by concomitant traumatic brain injury (TBI). However, since less than five years, it has been noted that individuals with SCI suffer from accelerated cognitive aging, even when correcting for mood factors and in the absence of TBI [1–4]. Individuals with SCI face a 13-fold increase in the risk of cognitive impairment compared to the general population [5] and they are twice as likely to develop Alzheimer’s dementia [6–8]. The cognitive domains most affected following SCI are executive functions, long-term memory, short-term memory, attention [2], processing speed and verbal fluency [1] when controlling for mood factors. This cognitive impairment begins in the subacute phase and seems to worsen over time [9]. It was found that individuals with SCI have cognitive impairments and brain activation patterns that are similar to healthy adults that are on average 20 years older [1, 10]. A proposed mechanism for this cognitive decline is neuroinflammation [4, 11]. Neuroinflammation plays a role in normal age-related cognitive decline [12–14] and following spinal injury, chronic elevation of neuroinflammation is found in the whole central nervous system [15–17].

Physical exercise interventions have been shown to have anti-inflammatory effects, induce an elevation of neurotrophic factors in the bloodstream, potentiate neuroplastic processes in the brain, increase brain volume and benefit cognitive function [18–21]. The anti-inflammatory effect and increase in neurotrophic factors during exercise results in part from the release of products from contracting muscle cells (i.e. myokines) [20]. This release of myokines has been reported following all sorts of muscle training, including electrical stimulation [20, 22]. It is often difficult for persons with SCI to sufficiently engage in physical exercise, with only 50% of SCI patients engaging in any leisure-time physical activity at all [23]. This difficulty may depend on the level of injury and the severity of motor function loss, as well as the availability of sport opportunities for wheelchair users. Electrical stimulation has been proposed as an interesting addition or alternative for increasing circulating myokine levels in humans with difficulty to participate in regular exercise programs [22]. Electrical stimulation can be used alone, i.e. neuromuscular electrical stimulation (NMES), or to assist voluntary exercise, i.e. functional electrical stimulation (FES). NMES induces contraction of myocytes similarly to exercise, which results in the release of myokines in the circulation, as recently reviewed by Sanchis-Gomar et al. (2019) [22]. A widely studied myokine is brain-derived-neurotrophic factor (BDNF), which is released from neurons [24], astrocytes [25], platelets [26], and skeletal muscle [27] and can trigger neuroprotective and neurotrophic effects [28]. Some studies suggest that most of the circulating BDNF is released from the brain [29]. Some other myokines, like insulin-like growth factor-1 and irisin were suggested to induce the release of brain-derived BDNF [20]. Remarkably, one study in young healthy adults reported that circulating levels of the myokine named BDNF increased more following a single bout of NMES than voluntary exercise with the same integrated force of muscle contraction [30]. Similarly, in a rat study, assessing able-bodied rats, the increase in BDNF levels was two times higher after four weeks of NMES compared to four weeks of running [31]. Also in human studies, long-term interventions with NMES were reported to increase circulating levels of BDNF, for example in older adults with type 2 diabetes [32]. BDNF can be considered one of the most important exercise-induced factors as it has direct beneficial effects on synaptic plasticity and neurogenesis, and it was linked with brain atrophy and cognitive function in a wide array of research [33]. So far, however, we were unable to find any studies reporting a beneficial effect of NMES on cognitive function. To the best of our knowledge, only one other study has evaluated the effects of FES [34] and only one study evaluated the effects of NMES [35] on cognitive function. The FES study is also the only study investigating exercise effects on cognition in SCI patients. In this study, improvements on a working memory task were reported after 6 months of functional electrical stimulation (FES)-assisted rowing exercise in SCI patients. This study did not measure myokine levels [34]. The authors of the NMES study evaluated the effect of a short intervention of 15 days of NMES in coronary bypass patients and discovered that patients in the experimental group showed increases in functional connectivity in the brain frontoparietal, salience and sensorimotor networks [35]. Overall, there is a lack of knowledge on the effects of electrical stimulation to improve cognitive function and the dose–response needed to attain such effect.

We will use a randomized replicated ABC single case experimental design (SCED) [36]. Primarily, we will examine to what extent and after how many weeks a 12 week intervention with NMES may change performance of people with SCI on the momentary digital symbol substitution task, an information processing task that will repeatedly be administered via a smartphone application. NMES will be applied to the quadriceps muscles of individuals with SCI. An intervention of 12 weeks is considered to be sufficient to induce both a myokine response and muscular changes. Secondarily, we will assess changes in the myokine BDNF before and after the 12 week intervention with NMES and after a 12 week follow-up without NMES. We hypothesize that NMES will have a beneficial effect on cognitive performance starting from the first weeks and that 12 weeks of NMES will induce an elevation of BDNF levels. This study tests a new treatment strategy, accessible even to individuals with low physical exercise possibilities, with the potential to slow down or prevent further cognitive decline in persons with SCI. The explorative findings may guide future research. Furthermore, it may help to raise awareness of the process of accelerated cognitive decline in persons with SCI.

## Methods

The Standard Protocol Items: Recommendations for Interventional Trials (SPIRIT) guidelines (see Appendix) and RoBiNT scale for risk of bias in SCED studies were used in designing and describing this clinical trial [37, 38].

The trial was registered with ClinicalTrials.gov (NCT05822297) on the 12th of January 2023, sponsored by Adelante Zorggroep and approved by the Medical Ethical Testing Committee (reference number W23.071) of Maxima Medical Center at Veldhoven, the Netherlands on the 9th of October 2023. In case of important protocol modifications, they will be notified to the Medical Ethical Testing Committee and updated in the trial registry.

### Participants

Participants and recruitment: Participants will be recruited from the outpatient clinic of the rehabilitation center Adelante Centre of Expertise in Rehabilitation and Audiology, locations Hoensbroek and Maastricht University Medical Center (MUMC +)., the Netherlands. The rehabilitation physician will inform potential participants about the study and flyers with information will be handed over. The flyers will include the contact details of one of the researchers. Alternatively, participants can give permission to the physicians that their contact information can be sent to the researchers.

Inclusion criteria: Participants are eligible if they are 18 years or older, have SCI since at least one year (chronic phase), the injury level is L2 or higher (meaning that the quadriceps muscle is likely affected to some extent, since this muscle is innervated by radicular nerves L3-L4), completeness of injury has been scored according to the American Spinal Injury Association (ASIA) Impairment Scale (AIS) [39] as either A, B or C, if they are able to use apps on their smartphones, if they are able to use NMES device safely at home, and if they speak Dutch at a native level. We will exclude participants if we cannot induce visible or palpable contractions of the quadriceps muscles with NMES or if they cannot tolerate the NMES intervention. We will exclude participants with a diagnosis of cancer, neurodegenerative or psychiatric disorders, current pressure ulcers, a history of severe autonomic dysreflexia (systolic blood pressure elevations above 200 mmHg), have metal implants in the stimulation site, an intrathecal baclofen pump, or are currently pregnant. Finally, we exclude participants who have taken part in a program with electrical stimulation in the last 6 months prior to the study.

### Study design and procedures

#### Study design

This study will apply a randomized replicated sequential ABC single-case experimental design (SCED), with a baseline (A), intervention (B) and follow-up (C) phase [36, 40]. SCED study designs demonstrate strong internal validity to determine the likelihood of a causal relationship between the intervention and outcomes. One entity is observed repeatedly over a certain time period under different levels of at least one independent variable. The power in SCEDs is related to the number of data points for each participant and not the number of participants. Each participant serves as his/her own comparison, thus controlling for confounding variables that can impact outcome and allowing heterogeneous clinical presentations. The internal validity of the SCED results can be improved by randomization techniques such as randomization of the starting time of the intervention. The downside is that the generalizability of the study results to the total population still depends on the number of participants included (i.e. replication of the findings) [40, 41]. The SCED lend itself perfectly to reflect on the reasons for the (likely) variability in the onset of NMES-induced effects on cognitive performance, looking at individual participant’s intervention courses. Thus, allowing to reflect on the existence of different potentially moderating characteristics. Several potential moderators have been suggested previously [4] and will be measured within this study. These include concomitant traumatic brain injury, psychological alterations, chronic pain, hemodynamic dysregulation, level of injury, respiratory failure, substance (ab)use, educational level, cardiovascular diseases, and intensive care admission [4]. In addition, a SCED design allows us to interpret after how many weeks the NMES leads to changes in performance on the momentary digital symbol substitution task. The aforementioned advantages of SCED studies indicate how this design offers additional information that is well fitted for an explorative study in a new topic.

In the context of SCED studies, randomization does not refer to individuals being randomly allocated to treatment groups, but to the random onset of the intervention phase which is usually set within a fixed window of time in order to make this randomization more feasible [38]. In this study, a window of 9 to 24 measurement points (i.e. 3 to 8 weeks at the set frequency of three test assessments per week) will be employed in which the change of phase from baseline to intervention phase will be randomly determined. This determination will be done with a random number generator in Excel set to give a random number between 9 and 24. There will be a baseline phase ranging from 3 to 8 weeks, followed by an intervention phase of 12 weeks and a final follow-up phase of 3 weeks after a 12 week rest period with no measurements and no interventions. As we will exclude the first 4 measurements in the baseline phase from analysis, see Sect. "Statistical analysis", this leaves us at least 5 measurement points in this phase, 36 measurement points in the intervention phase and 9 measurement points in the follow-up phase. Neither participants nor researchers will be blinded to the phase of the intervention. Replication of the experiment will be done in 15 participants.

Finally, additional measurements will be done in a repeated manner according to a single-armed prospective design before baseline phase, at the end of the baseline phase, at the end of the intervention phase and at the start of the follow-up phase.

#### Study procedures

**Fig. 1:**
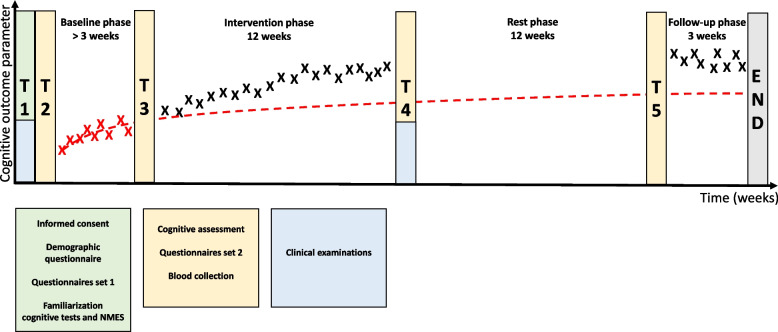
Experimental design. T1-5 represent measurement time points. During T2-5 the same measurements are repeated, expect the addition of clinical examination in time point T4. The red line is the expected change in cognitive test performance on the smartphone-based cognitive test without intervention, which we expect to show a fast increase in the first ± 3 sessions followed by a slight increase over time due to a learning effect. The X’s mark how cognitive test results are hypothesized to change due to the intervention

The experimental design is illustrated in Fig. 1. Upon the first appointment (T1), participants will sign an informed consent and undergo baseline examinations consisting of questionnaires and clinical tests. A smartphone application for repeated monitoring of cognitive function will be installed on their smartphones and they will be familiarized with the cognitive tests and neuromuscular electrical stimulation intervention. On a separate day (T2), within seven days from the first appointment, they will undergo a series of cognitive tests and venous blood samples will be collected. Furthermore, they will fill in a second list of questionnaires and undergo a clinical evaluation of strength and spasticity. This will be followed by a no-treatment baseline period with a random length of 3–8 weeks. Upon the third (T3), fourth (T4) and fifth (T5) visit, the oral cognitive tests, venous blood sample collection and questionnaires from T2 will be repeated. During the third visit (T3) the participants will receive their own NMES device for home-based training and they will be asked to return it 12 weeks later on the fourth visit (T4). All participants will undergo the same intervention. During the fourth visit (T4) the clinical evaluation of strength and spasticity that was done at T2 will be repeated. Between T2 and T4 and between T5 and T6 participants will undergo smartphone cognitive tests 3 times per week. At the end of the study, no additional tests will be done. Participants will be requested to write in a diary the specifications of the NMES session (duration, intensity) after each session and indicate when they performed a cognitive test. They can also write down any problems they encountered using the NMES device or smartphone application. Participants receive a telephone call once a week by one of the researchers to assess how they feel, be reminded of the diary and to assure there are no technical issues with the NMES device or smartphone application. Participants are allowed to withdraw from the study at any time. Available data from these participants will be included in the data analyses whenever possible.

### Intervention

**Fig. 2:**
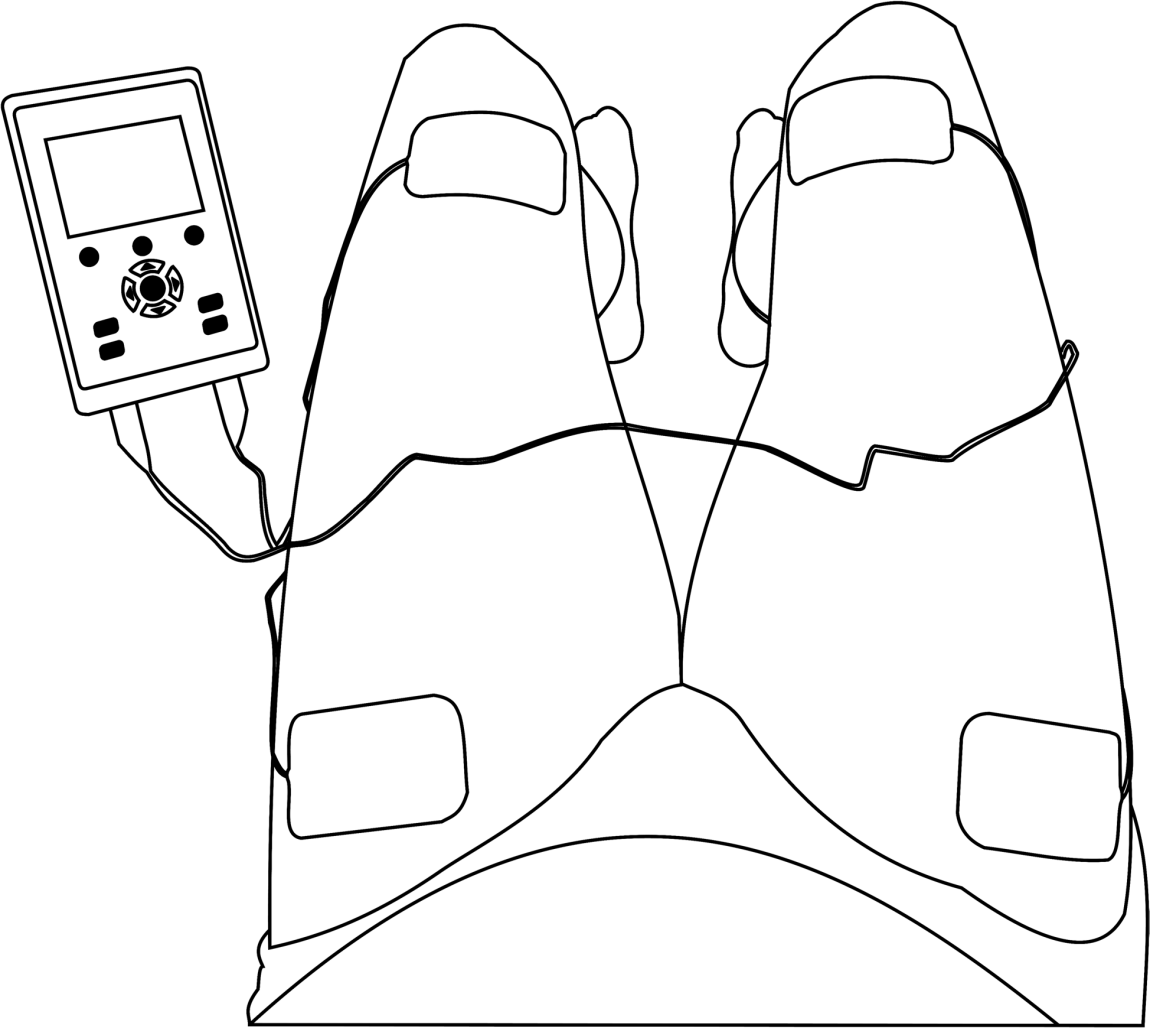
Placement of the electrodes on the quadriceps muscles

The intervention consists of 12 weeks NMES which will be done using the Pierensymphony M, serie A, article number 104800 (10,003,566) (manufactured by Pierenkemper GmbH and distributed by schwa-medico Nederland B.V.), a two-channel NMES device designed, and CE marked for muscular electrical stimulation (CE 0482). After a familiarization session, the intervention will be home-based, three times a week, with at least 1 day between stimulation sessions and 2 days between a stimulation session and testing day. Electrical stimulation will be done on the quadriceps muscles of both legs simultaneously. For each leg, one electrode is placed on the proximal lateral side and one on the distal medial side of the quadriceps muscle (see Fig. 2). Electrical stimulation will take 30 min, at a stimulation frequency of 50 Hz, and a pulse width of 400 μs. We will choose the highest intensity that is easily supported by the participant without inducing discomfort with a maximum intensity of 100 mA. We should at least see a visible or palpable contraction or the participant will be excluded. The activation within the activation-rest cycle consists of a 1 s ramp-up, 7 s full activation and 1 s ramp-down, followed by 18 s rest. Every 4 weeks the rest period will be diminished with 3 s until the rest phase is equal to the activation phase (9 s). Participants may continue their normal treatments, which may consist of physiotherapy. However, they will be asked not to change their physical activity habits during the experiment.

### Cognitive assessments

#### Smartphone-based cognitive assessment

To study the primary aim of our study, participants will undergo a cognitive test for 3 times a week during the baseline, intervention and follow-up phase of the experiment. This repetitive cognitive test will be administered using a secured smartphone application, which was designed for use in clinical settings and research (m-Path, https://m-path.io/landing/). It will be programmed to emit an auditory signal three times a week signaling the availability of a new cognitive test between 7.30 AM and 10.30 PM on the days that participants do not undergo NMES sessions. The test is the momentary digital symbol substitution task, which is a measure of processing speed and short-term working memory function. For this test, participants have 30 s time to complete as many trials as possible where they need to correctly select the figure representing the number given as depicted in the legend provided on the top of the screen. The outcome is presented as response time derived from the number of trials/30 s (speed, in ms) and the percentage of correct trials (accuracy). Before the test starts, participants will be informed that they should be ready to respond as fast and accurate as possible. Daniëls et al. (2020) have validated this test for repeated use in healthy adults. They reported a significant learning effect for number of correct answers in 30 s between the first and 48th trial (B = 0.32, SE = 0.04, *p* < 0.001; with number of correct answers as the dependent variable and the logarithmic transformation of the session number (between 1 and 48) as the independent variable) [42].

#### Oral cognitive test battery

At four time points, participants will undergo an oral cognitive test battery consisting of seven tests that were validated for repeated use in people with SCI, even with impaired hand function [43]. The Dutch version of these tests will be used, whenever the test depends on language. The tests have a good-to-excellent test–retest reliability, except the RAVTL recognition score which has been shown to have a poor test–retest reliability [43]. This test was still used as the interference recall and delayed recall scores of this test have a good test–retest reliability in people with SCI [43]. To further decrease learning effects of repeated administration of the cognitive tests, different versions will be used on different visits if available. The test versions will be numbered and on their first visit participants will be asked to draw a random number from an envelope including a number for every existing version of the cognitive test, until the envelope is empty. This is repeated for every cognitive test. The sequence will be reported for the next visits. The cognitive tests are presented in Table 1.

**Table 1:** Oral cognitive assesments

Cognitive test	Description
**Information processing speed**	
SDMT	This test is similar to the m-Path smartphone test. However, now, the participant is asked to verbally match the number representing the symbol given as depicted in the legend provided on the top of the page. The researcher writes down the answers on a separate page. The first 10 trials are used for familiarization, then the participant is given 90 s to verbally match as many numbers to symbols as possible. The final score is obtained by subtracting the number of errors from the number of items completed within the given timeframe [34]. To minimize learning effects, three different versions of this test will be used
**Verbal fluency**	
COWAT	For this test participants are asked three times to name as many words starting with a specific letter in one minute time. Words cannot be named more than once and non-existing words are not counted. In addition, names of persons and numbers are also not allowed. There are three versions of this test (DAT, KOM, PGR) in the Dutch language. The total score is the sum of the number of words named for each of the three letters [34, 35]
**Attention/concentration**	
Digit Span Forward	Participants will be given a series of numbers, starting with 2 numbers and progressing to 9 numbers, read by an assessor. Participants will be asked to repeat the numbers in the same order. They get two trials before progressing to a larger series of numbers. When both series are answered incorrectly, the test is ended. The total score is the number of correctly answered series [34]. There are two versions of this test available at our institution
**Executive functions**	
Digit Span Backward	This version of the digit span adds a working memory component to the test when compared to the forward digit span. Participants will be given a series of numbers, starting with 2 numbers and progressing to 9 numbers, read by an assessor. Participants will be asked to repeat the numbers backwards. They get two trials before progressing to a larger series of numbers. When both series are answered incorrectly, the test is ended. The total score is the number of correctly answered series [34]. There are two versions of this test available at our institution
TMT A & B	This test assesses switching ability. This test encompasses two parts. In trial A, the participant verbally count from 1 to 25 as quickly as possible. In trial B, the participant will be instructed to verbally alternate between numbers and letters of the alphabet until 13. The time difference between trial A and B will be recorded. This test lasts approximately 3 min [34, 36]
Stroop test	This test assesses cognitive inhibition, but also attention and processing speed. This test consists of three lists that the participant must read out loud; the first list has the names of colors printed in black ink, the second list with the colors printed in colored ink, and the final list having the name of colors printed in incongruent colored ink (e.g., the word red is printed in blue ink). The time to complete second list, third list, as well as the time difference between these latter two lists will be recorded. The test takes approximately 5 min. There is one version of this test available [34, 37]
**Memory**	
15 word test (RAVLT)	The 15 word test is the RAVLT. This test consists of a list of 15 words that is read to the participant by an assessor. After reading, the participant must try to recall as many words as they can. This read and recall process is repeated an additional four times. A distractor list (second list) is presented with a recall attempt, followed by a timed recall of the first list. The timed recall measures the participant’s ability to recall information despite the intervening list. After 10 min, the participant performs another timed recall of the first list of words. Afterwards the participant is given a recognition task where they identify the 15 words from the initial list from a paragraph containing 30 underlined words. This final trial distinguishes memory storage from inefficient recall [34]. There are two versions of this test available at our institution

Abbreviations: *COWAT* controlled word association test, *RAVLT* Rey Auditory Verbal Learning Test, *SDMT* Symbol Digit Modalities Test, *TMT* Trail Making Test

### Blood analysis

Venous blood samples will be taken at the antecubital vein. Blood samples will be collected in two 5 mL serum separator tubes. After blood collection, the tubes will be gently inverted 6–8 times and within 90 min be centrifuged. Quantitative determination of serum BDNF will be measured using enzyme-linked immunosorbent assays (ELISA). Blood will be drawn four times (T2-5) per participant during the whole study project. To decrease errors due to ELISA kit differences, we will store the blood samples in a refrigerator compartment at the Maastricht University laboratory site at -80 °C until analysis, using the same kit for all blood measurements of the same participant at the end of the project.

### Demographic and clinical assessments

At baseline, we will collect information on participant’s age, gender, smoking status, years of education, cause of the SCI, date of the SCI, drug/alcohol use, body mass index (BMI), medical history such as diabetes mellitus or hemodynamic regulation problems (hypertension, hypotension, autonomic dysreflexia) or respiratory problems (nocturnal apnea or use of a device for sleep apnea, signs of restricted or obstructive lung disease on spirometry test) or history of intensive care admission, medication use, history of TBI, and infections or wounds in the past 3 weeks. Some clinical tests will also be conducted upon baseline, while others are also measured at follow-up since we expect they may change due to our intervention. The latter are therefore seen as secondary outcomes and may be evaluated in a separate study.

The following clinical tests will be conducted:

At measurement timepoint 1

- SCI classification: Functional impairment in terms of sensation and strength of key muscles will be assessed using the AIS classification [39]. It is a structured clinical assessment of the level and completeness of the spinal cord injury. Intra- and interobserver correlation coefficients are generally around 0.9 [44].
- Lung spirometry: A portable spirometer (Vitalograph In2itive) will be used to assess forced expiratory volume in one second (FEV1), forced vital capacity (FVC) and peak cough flow (PCF) (L/s).
- Heart rate and blood pressure measurement using an automated device will be measured at the left or right arm in seated position.

At measurement timepoint 1 and 4

- Spasticity level: The Perceived Resistance to Passive Movement (PRPM) test is recommended by the Dutch guidelines for measuring spinal spasticity [45]. The scale ranges from (0) ‘no increased resistance’ to (4) ‘movement of the limb impossible’. Resistance of elbow flexors, elbow extensors, wrist flexors, wrist extensors, hip adductors, knee flexors, knee extensors, ankle plantar flexors (both with extended and with a 90° flexed knee) will be assessed in supine position. A sum of the scores will be used as the outcome measure, ranging from 0 to 32.
- Electrically-evoked muscle strength: The MicroFET2 (Hoggan Scientific, LLC) muscle strength testing system is an handheld dynamometer, which will be used to measure electrical stimulation-evoked quadriceps force. The electrical stimulation protocol will be equal to that used during the 12 week intervention. The dynamometer will be affixed to a band, which in turn will be secured around the wheelchair and the distal part of the participant's lower leg. The MicroFET2 dynamometer demonstrates a high intra-rater reliability for knee extension, with an interclass correlation coefficient (ICC) of 0.93, a standard error of measurement (SEM) of 17.2N and a minimal detectable change of 47.5N [46].

The following questionnaires will be used:

At measurement timepoint 1

- Brief Physical Activity Assessment Tool: This tool is a two question physical assessment. It has a validity similar to that of more detailed self-report measures of physical activity. It can be used efficiently in routine primary healthcare services to identify insufficiently active patients who may need physical activity advice [47].
- TBI-4 questionnaire: This questionnaire is specifically designed as a self-report tool to determine the likelihood of TBI in traumatic SCI patients utilizing just two questions. The outcome is presented as improbable TBI, possible TBI, mild TBI, moderate TBI or severe TBI. At the cut-off “possible TBI” this tool had a sensitivity of 83% and a specificity of 51% to detect mild TBI based on medical reports, indicating this questionnaire can be used as a guide in the unavailability of medical reports, but should be interpreted with care [48].

At measurement timepoints 2 till 5

- Cognitive Failures Index: This test can reliably evaluate how participants experience their own cognitive function. It consists of 25 questions, each scored on a 5-point scale ranging from never (0) to very often (5) [49].
- McGill Pain Questionnaire: Participants will be given a figure of a person, where they can color the zone of pain. They will be asked to describe the pain and score it on a 10 point visual analogue scale (VAS) and will receive some questions related to the impact of pain on their daily life [50].
- Hospital Anxiety and Depression Scale (HADS): this scale is a widely used and reliable screening instrument to assess the severity of anxiety and depression [51]. A subtest score of more than 8 on 21 denotes considerable symptoms of anxiety or depression.
- Fatigue Severity Scale: This questionnaire encompasses nine questions and has previously been used to recognize and diagnose fatigue in patients with neurological disorders [52].
- Pittsburgh Sleep Quality Index: This index consists of 19 questions concerning seven domains related to sleep quality in the preceding month. Every domain receives a score of 0–3, with a total score of 0–21. A score of more than 5 indicates a poor sleep quality [53].
- Utrecht Scale for Evaluation of Rehabilitation – Participation (USER-P): This test is widely used to assess participation in daily life activities in rehabilitation contexts. It contains 32 questions related to the frequency of participation, participation restrictions and satisfaction with participation, with a total score of 0–100 for each of these three domains. A higher score indicates a better participation [54].

The baseline measurements of potential moderators of the effect of our intervention on cognitive and blood outcomes and risk factors for cognitive impairments according to a previous review paper will be entered in the statistical analysis [4]. The risk factors for cognitive impairments will be entered as a combined risk score with a maximum score of 10, indicating the highest risk. Participants receive a score between zero and one for each of the following: 1 point if self-reported history of TBI or mild to severe concomitant TBI according to TBI4 questionnaire [48], 0.5 points for a score of > 8/21 for anxiety and 0.5 points for a score of > 8/21 for depression on the HADS scale [51], 1 point for chronic pain indicated on the McGill pain questionnaire to exist longer than 3 months and is scored at its minimum a VAS score of 3 [50], 0.5 points for a history of autonomic dysreflexia and 0.5 points for a blood pressure below 90/60 upon measurement, 1 point for tetraplegic participants compared to 0 points for paraplegic participants, 0.33 points for self-reported sleep apnea, 0.33 points for a FVC value below 0.85% of the predicted value, and 0.33 points for a PCF below 4.5L/s, 0.25 points for regular alcohol use (more than once per week), 0.25 points for drug abuse, 0.25 points for polypharmacy (more than 7 prescribed drugs), 0.25 points for any medication acting on the central nervous system, 1 point for participants who finished only basic education, 0.5 points for participants who finished only secondary education, 0.5 points for diabetes mellitus, 0.5 points for a BMI equal to or above 25 kg/m^2^ [55], 1 point for previous intensive care admission.

### Statistical analysis

#### Single-case data

For our primary aim, the smartphone cognitive assessment, statistical analysis will be performed using the Shiny app for Single-Case Data Analysis (Shiny SCDA) [56]. A total of 54–69 measurements per participant will be attained.

##### Visual analysis

First, measurement points will be plotted and visually inspected per participant. Six features will be visually examined according to the guidelines of Lane and Gast (2014): cognitive performance in the different phases, variability in cognitive performance both within and between phases, trends in the data, immediacy of effect, overlap of data points between phases, and consistency of data patterns across participants [57]. Instead of inspecting only immediate effects, also the existence of a potential delay of the effect and consistency of this delay between participants will be explored [58].

##### Effect size measures

Finally, effect size measures will be calculated for each participant. This will be done by calculating the percentage of non-overlapping pairs between phases [59]. The NAP (Nonoverlap of All Pairs) value equals the number of comparison pairs showing no overlap, divided by the total number of comparisons, and can be considered an area under the curve percentage from a receiver operating characteristic analysis [60].

##### Randomization test

Subsequently, randomization tests will be performed to test the null hypothesis that NMES does not have an effect on participant’s cognitive function. The observations of the baseline phase will be compared to those of the intervention and follow-up phases respectively. The test statistic “means of phase A minus means of phase B” will be chosen as the primary outcome of this study. In case visual analysis suggests delayed effects, the randomization test will be repeated with lagged data until the lowest p-value has been reached (one effect lag equals one day). The first 4 measurements in the baseline phase will be removed before calculating the means, since we expect a learning effect on the first trials, leaving between 5 and 20 measurement points depending on the randomly decided length of the baseline period. Studies that tested the statistical properties of a randomization test used in this type of design showed that the Type I error probability of the randomization test was maintained at an acceptable level [61].

##### Missing data

Missing values from the repeated smartphone-based cognitive test will be treated with the randomized marker method [62]. In this method, the missing value is removed from calculation of the mean in the randomization test. In addition, the position of the missing value will be randomly reshuffled within possible randomization schemes of the study protocol. In this study, this means that a missing value in the baseline or beginning of the intervention phase may be reshuffled to be part of the other phase if this falls within the possibilities (i.e. if it falls within the first 4 to 8 weeks of the study). This method was found to be more effective at controlling type I error and resulted in higher power than multiple imputation and single imputation using a time series model in a SCED simulation study [63].

#### Secondary data

For the secondary study parameters (i.e. BDNF levels and results of the oral cognitive test battery) which are measured within a single-armed prospective design, statistical analysis will be done using IBM SPSS Statistics 27.

##### Secondary statistics

Before analysis, the data will be checked for outliers, defined as values lying further than three times the interquartile range away from the median value. The normality assumption will be checked based on visual representations of the data using histograms and measurements of skewness and kurtosis (normality assumed if the data values lie between -2 and + 2) [64]. Homoskedasticity will be tested using the Levene’s test. Descriptive statistics will be used. Furthermore, the Friedman test will be used for analysis of repeated measurements. The False Discovery Rate (FDR) method will be applied to correct for the multiple testing problem. In the FDR method, every *p*-value is compared against a sequentially weighted threshold on all *p*-values [65].

##### Missing data

Missing pre-post values will not be replaced by specific values, but be excluded from statistical analysis.

### Sample size calculation

#### Single-case data

The sample size needed for the abovementioned statistical analyses is different for the SCED and single-armed prospective study design. For the SCED design, the minimum sample size is *n* = 1. Instead, the power of the analysis depends on the number of observations [40, 41]. For randomization tests the lowest attainable *p*-value is calculated by dividing 1 by the number of possible permutations. In this study, the baseline phase, after removing the first four values because of a learning effect, can contain 5 to 24 measurement points, allowing 20 possible permutations. This corresponds with a lowest attainable *p*-value of 0.05 [56]. Of note, successful replication of the single-case experiment in additional participants with similar symptoms will improve the generalizability of the results [40, 41].

#### Secondary data

For the single-armed prospective study design we have estimated the sample size needed in order to have sufficient power (Power = 0.80) for evaluating a Time effect (over 4 time points) of cognitive test performance changes with repeated measures ANOVA. We found no previous studies examining the effect of any muscle activity intervention in spinal cord injury subjects. Therefore, the required effect size was estimated to be similar to that from a meta-analysis examining the effect of resistance exercise interventions on general cognitive function in healthy adults [66]. The overall effect size (Cohen’s d) was 0.71 (0.30–1.12) for resistance exercise. The correlation among repeated measures for this cognitive test battery that was found to be ≥ 0.77 in a previous SCI study [43]. G*Power 3.1.9.7 estimated that the minimum total sample size should be 7. Taking into account potential drop-outs and the uncertainty of the effect, we decided to aim for a total sample of 15 included participants.

### Data management

All the participant data will be coded and pseudomized to protect their privacy. The repeated cognitive test will be administered using a secured smartphone application (see www.m-path.io/landing), which was designed for use in clinical settings and research and is General Data Protection Regulation (GDPR) compliant. Participants will register to m-Path with their study code instead of their personal name. With a password, one of the researchers can access a dashboard where data from all included participants will be visible. Other test results including participant identification details will be stored at Adelante Hoensbroek in a locked cabinet with restricted access. The key will be kept in another locked cabinet. The test results with participant identification will only be accessed again after coding and de-identifying the data in order to hand them over to participants requesting their test results. The data will be stored for 15 years. Blood samples will be destroyed after analysis, which is expected to take place within one year after blood collection. We intend to make pseudomized data from our study available according to the FAIR principle, such that it is Findable, Accessible, Interoperable and Reusable. Both negative and positive results will be made public. Our results will be presented on the Dutch national rehabilitation medicine congress and international rehabilitation/neuroscience congresses so that rehabilitation professionals are more aware of the benefit of muscle training or maintaining a healthy body overall on the brain and cognitive function, and so that neuroscientists will understand the relevance of neuromuscular electrical stimulation as one of the muscle training strategies to investigate the exercise-cognition link. The results will be conveyed locally to spinal cord injury subjects in our rehabilitation center in Hoensbroek and in the Maastricht University Medical Center (MUMC +). We will inform other national spinal cord injury clinics of the results and we will discuss the findings in the national work group of the Dutch rehabilitation society for movement and sport. Finally, the results will be submitted to a peer-reviewed journal.

#### Risks and harms

The use of electrical stimulation is generally safe. It has even been used unsupervised in spinal cord injury subjects during sleep [67]. However some adverse events or inconveniences that have been reported previously in literature are a red, raised or itchy skin; muscle pain; increased neuropathic pain; uncomfortable feeling; orthostatic hypotension (dizziness, light-headedness, blurred vision, palpitation or shortness of breath); in some cases pain from a spasm may occur. The adverse events reported are temporary added risks that disappear once the stimulus/stimulation has stopped [68]. In case the subject feels uncomfortable during electrical stimulation, they will be informed that the stimulation can be interrupted. Participants with a lesion level above T6 may experience autonomic dysreflexia in response to the electrical stimulation. Whenever this occurs, stopping the electrical stimulation should solve the problem. Participants with lesion levels above T6 will be explained how they can recognize signs of autonomic dysreflexia. Whenever autonomic dysreflexia has occurred during the NMES training period participants are asked to contact the medical professional who’s telephone number is provided to them at the beginning of the project and in the participant information. The sponsor has a liability insurance which covers for damage to research participants. All adverse events reported spontaneously by a participant or observed by the investigator will be recorded.

### Supplementary Information

Supplementary Material 1.

## Data Availability

Not applicable (this manuscript does not report data generation or analysis). I have indicated not applicable, but I keep getting messages after technical check that a statement is missing. Therefore, I indicated 'Yes' now and added in this text box that it is not applicable.

## References

[1] Chiaravalloti ND, Weber E, Wylie G, Dyson-Hudson T, Wecht JM (2020). Patterns of cognitive deficits in persons with spinal cord injury as compared with both age-matched and older individuals without spinal cord injury. J Spinal Cord Med.

[2] Heled E, Tal K, Zeilig G (2020). Does lack of brain injury mean lack of cognitive impairment in traumatic spinal cord injury?. J Spinal Cord Med.

[3] Li Y, Cao T, Ritzel RM, He J, Faden AI, Wu J (2020). Dementia, depression, and associated brain inflammatory mechanisms after spinal cord injury. Cells.

[4] Vints WAJ, Levin O, Masiulis N, Verbunt J, van Laake-Geelen CCM (2023). Myokines may target accelerated cognitive aging in people with spinal cord injury: a systematic and topical review. Neurosci Biobehav Rev.

[5] Craig A, Guest R, Tran Y, Middleton J (2017). Cognitive impairment and mood states after spinal cord injury. J Neurotrauma.

[6] Huang SW, Te WW, Chou LC, Liou TH, Lin HW (2017). Risk of dementia in patients with spinal cord injury: a nationwide population-based cohort study. J Neurotrauma.

[7] Yeh T-S, Ho Y-C, Hsu C-L, Pan S-L (2018). Spinal cord injury and Alzheimer’s disease risk: a population-based, retrospective cohort study. Spinal Cord.

[8] Mahmoudi E, Lin P, Peterson MD, Meade MA, Tate DG, Kamdar N (2021). Traumatic spinal cord injury and risk of early and late onset Alzheimer’s disease and related dementia: large longitudinal study. Arch Phys Med Rehabil.

[9] Molina B, Segura A, Serrano JP, Alonso FJ, Molina L, Pérez-Borrego YA (2018). Cognitive performance of people with traumatic spinal cord injury: a cross-sectional study comparing people with subacute and chronic injuries. Spinal Cord.

[10] Wylie GR, Chiaravalloti ND, Weber E, Genova HM, Dyson-Hudson TA, Wecht JM (2020). The neural mechanisms underlying processing speed deficits in individuals who have sustained a spinal cord injury: a pilot study. Brain Topogr.

[11] Alcántar-Garibay OV, Incontri-Abraham D, Ibarra A (2022). Spinal cord injury-induced cognitive impairment: a narrative review. Neural Regen Res.

[12] Sartori AC, Vance DE, Slater LZ, Crowe M (2012). The impact of inflammation on cognitive function in older adults: Implications for healthcare practice and research. J Neurosci Nurs.

[13] Cleeland C, Pipingas A, Scholey A, White D (2019). Neurochemical changes in the aging brain: a systematic review. Neurosci Biobehav Rev.

[14] Vints WAJ, Kušleikienė S, Sheoran S, Šarkinaitė M, Valatkevičienė K, Gleiznienė R (2022). Inflammatory blood biomarker kynurenine is linked with elevated neuroinflammation and neurodegeneration in older adults: evidence from two 1H-MRS post-processing analysis methods. Front Psychiatry.

[15] Li F, Huo S, Song W. Multidimensional review of cognitive impairment after spinal cord injury. Acta Neurol Belg. 2020;121(1):37–46. 10.1007/s13760-020-01507-y 32989706

[16] Sun X, Jones ZB,  Chen XM, Zhou L, So KF, Ren Y (2016). Multiple organ dysfunction and systemic inflammation after spinal cord injury: A complex relationship. J Neuroinflammation.

[17] Wu J, Zhao Z, Kumar A, Lipinski MM, Loane DJ, Stoica BA (2016). Endoplasmic reticulum stress and disrupted neurogenesis in the brain are associated with cognitive impairment and depressive-like behavior after spinal cord injury. J Neurotrauma.

[18] Erickson KI, Hillman C, Stillman CM, Ballard RM, Bloodgood B, Conroy DE (2019). Physical activity, cognition, and brain outcomes: a review of the 2018 physical activity guidelines. Med Sci Sports Exerc.

[19] Mee-Inta O, Zhao ZW, Kuo YM (2019). Physical Exercise Inhibits Inflammation and Microglial Activation. Cells.

[20] Vints WAJ, Levin O, Fujiyama H, Verbunt J, Masiulis N (2022). Exerkines and long-term synaptic potentiation: mechanisms of exercise-induced neuroplasticity. Front Neuroendocrinol.

[21] Sheoran S, Vints WAJ, Valatkevičienė K, Kušleikienė S, Gleiznienė R, Česnaitienė VJ, et al. Strength gains after 12 weeks of resistance training correlate with neurochemical markers of brain health in older adults: a randomized control 1H-MRS study. GeroScience. 2023;45(3):1837–55. 10.1007/s11357-023-00732-6 PMC9877502 36701005

[22] Sanchis-Gomar F, Lopez-Lopez S, Romero-Morales C, Maffulli N, Lippi G, Pareja-Galeano H (2019). Neuromuscular electrical stimulation: a new therapeutic option for chronic diseases based on contraction-induced myokine secretion. Front Physiol.

[23] Martin Ginis KA, Latimer AE, Arbour-Nicitopoulos KP, Buchholz AC, Bray SR, Craven BC (2010). Leisure time physical activity in a population-based sample of people with spinal cord injury part i: demographic and injury-related correlates. Arch Phys Med Rehabil.

[24] Lessmann V, Gottmann K, Malcangio M (2003). Neurotrophin secretion: current facts and future prospects. Prog Neurobiol.

[25] Numakawa T, Suzuki S, Kumamaru E, Adachi N, Richards M, Kunugi H (2010). BDNF function and intracellular signaling in neurons. Histol Histopathol.

[26] Le Blanc J, Fleury S, Boukhatem I, Bélanger JC, Welman M, Lordkipanidzé M (2020). Platelets selectively regulate the release of BDNF, But not that of its precursor protein, proBDNF. Front Immunol.

[27] Máderová D, Krumpolec P, Slobodová L, Schön M, Tirpáková V, Kovaničová Z (2019). Acute and regular exercise distinctly modulate serum, plasma and skeletal muscle BDNF in the elderly. Neuropeptides.

[28] Knaepen K, Goekint M, Heyman EM, Meeusen R (2010). Neuroplasticity - exercise-induced response of peripheral brain-derived neurotrophic factor: a systematic review of experimental studies in human subjects. Sport Med.

[29] Serra-Millàs M (2016). Are the changes in the peripheral brain-derived neurotrophic factor levels due to platelet activation?. World J Psychiatry.

[30] Kimura T, Kaneko F, Iwamoto E, Saitoh S, Yamada T (2019). Neuromuscular electrical stimulation increases serum brain-derived neurotrophic factor in humans. Exp Brain Res.

[31] Dalise S, Cavalli L, Ghuman H, Wahlberg B, Gerwig M, Chisari C (2017). Biological effects of dosing aerobic exercise and neuromuscular electrical stimulation in rats. Sci Rep..

[32] Miyamoto T, Iwakura T, Matsuoka N, Iwamoto M, Takenaka M, Akamatsu Y (2018). Impact of prolonged neuromuscular electrical stimulation on metabolic profile and cognition-related blood parameters in type 2 diabetes: a randomized controlled cross-over trial. Diabetes Res Clin Pract.

[33] Müller P, Duderstadt Y, Lessmann V, Müller NG (2020). Lactate and BDNF: key mediators of exercise induced neuroplasticity?. J Clin Med.

[34] Ozturk ED, Lapointe MS, Il Kim D, Hamner JW, Tan CO (2021). Effect of 6-month exercise training on neurovascular function in spinal cord injury. Med Sci Sports Exerc.

[35] Lo Re V, Russelli G, Lo Gerfo E, Alduino R, Bulati M, Iannolo G (2023). Cognitive outcomes in patients treated with neuromuscular electrical stimulation after coronary artery bypass grafting. Front Neurol.

[36] Krasny-Pacini A, Evans J (2018). Single-case experimental designs to assess intervention effectiveness in rehabilitation: a practical guide. Ann Phys Rehabil Med.

[37] Tetzlaff JM, Moher D, Chan AW (2012). Developing a guideline for clinical trial protocol content: Delphi consensus survey. Trials.

[38] Tate RL, Perdices M, Rosenkoetter U, Wakim D, Godbee K, Togher L (2013). Revision of a method quality rating scale for single-case experimental designs and n-of-1 trials: the 15-item risk of bias in N-of-1 Trials (RoBiNT) Scale. Neuropsychol Rehabil.

[39] Betz R, Biering-Sørensen F, Burns SP, Donovan W, Graves DE, Guest J (2019). The 2019 revision of the International Standards for Neurological Classification of Spinal Cord Injury (ISNCSCI)-what’s new?. Spinal Cord.

[40] Vlaeyen JWS, Onghena P, Vannest KJ, Kratochwill TR (2022). Single-case experimental designs: clinical research and practice. Compr Clin Psychol (Second Ed).

[41] Kazdin AE (2019). Single-case experimental designs. Evaluating interventions in research and clinical practice. Behav Res Ther..

[42] Daniëls NEM, Bartels SL, Verhagen SJW, Van Knippenberg RJM, De Vugt ME, Delespaul PAEG (2020). Digital assessment of working memory and processing speed in everyday life: Feasibility, validation, and lessons-learned. Internet Interv.

[43] Nightingale TE, Lim CAR, Sachdeva R, Zheng MMZ, Phillips AA, Krassioukov A (2019). Reliability of cognitive measures in individuals with a chronic spinal cord injury. PM R.

[44] Roberts TT, Leonard GR, Cepela DJ (2017). Classifications in brief: American Spinal Injury Association (ASIA) impairment scale. Clin Orthop Relat Res.

[45] Nederlandse Vereniging van Revalidatieartsen. Evaluatie van spasticiteit - Cerebrale en/of spinale spasticiteit. 2016. https://richtlijnendatabase.nl/richtlijn/cerebrale_en_of_spinale_spasticiteit/cerebrale_en_of_spinale_spasticiteit_-_startpagina.html. Accessed 26 Feb 2023.

[46] Jackson SM, Cheng MS, Smith AR, Kolber MJ (2017). Intrarater reliability of hand held dynamometry in measuring lower extremity isometric strength using a portable stabilization device. Musculoskelet Sci Pract.

[47] Marshall AL, Smith BJ, Bauman AE, Kaur S (2005). Reliability and validity of a brief physical activity assessment for use by family doctors. Br J Sports Med.

[48] Brenner LA, Homaifar BY, Olson-Madden JH, Nagamoto HT, Huggins J, Schneider AL (2013). Prevalence and screening of traumatic brain injury among veterans seeking mental health services. J Head Trauma Rehabil.

[49] Wassenaar A, De Reus J, Donders ART, Schoonhoven L, Cremer OL, De Lange DW (2018). Development and validation of an abbreviated questionnaire to easily measure cognitive failure in ICU survivors: a multicenter study. Crit Care Med.

[50] Melzack R (1975). The McGill pain questionnaire: major properties and scoring methods. Pain.

[51] Spinhoven P, Ormel J, Sloekers PPA, Kempen GIJM, Speckens AEM, Van Hemert AM (1997). A validation study of the Hospital Anxiety and Depression Scale (HADS) in different groups of Dutch subjects. Psychol Med.

[52] Rietberg MB, Van Wegen EEH, Kwakkel G (2010). Measuring fatigue in patients with multiple sclerosis: reproducibility, responsiveness and concurrent validity of three Dutch self-report questionnaires. Disabil Rehabil.

[53] Buysse DJ, Reynolds CF, Monk TH, Berman SR, Kupfer DJ (1989). The pittsburgh sleep quality index: a new instrument for psychiatric practice and research. Psychiatry Res.

[54] Van Der Zee CH, Post MW, Brinkhof MW, Wagenaar RC (2014). Comparison of the utrecht scale for evaluation of rehabilitation-participation with the ICF measure of participation and activities screener and the WHO disability assessment schedule II in persons with spinal cord injury. Arch Phys Med Rehabil.

[55] Laughton GE, Buchholz AC, Martin Ginis KA, Goy RE (2009). Lowering body mass index cutoffs better identifies obese persons with spinal cord injury. Spinal Cord.

[56] De TK, Michiels B, Vlaeyen JWS, Onghena P (2020). Shiny SCDA (Computer software).

[57] Lane JD, Gast DL (2014). Visual analysis in single case experimental design studies: brief review and guidelines. Neuropsychol Rehabil.

[58] Ledford JR, Lane JD, Severini KE (2018). Systematic use of visual analysis for assessing outcomes in single case design studies. Brain Impair.

[59] Parker RI, Vannest KJ, Davis JL (2011). Effect size in single-case research: a review of nine nonoverlap techniques. Behav Modif.

[60] Parker RI, Vannest K (2009). An improved effect size for single-case research: nonoverlap of all pairs. Behav Ther.

[61] Levin JR, Ferron JM, Kratochwill TR (2012). Nonparametric statistical tests for single-case systematic and randomized ABAB…AB and alternating treatment intervention designs: new developments, new directions. J Sch Psychol.

[62] De TK, Onghena P (2022). The randomized marker method for single-case randomization tests: Handling data missing at random and data missing not at random. Behav Res Methods.

[63] De TK, Michiels B, Tanious R, Onghena P (2020). Handling missing data in randomization tests for single-case experiments: a simulation study. Behav Res Methods.

[64] George D, Mallery P (2010). SPSS for windows step by step: a simple guide and reference, 17.0 update.

[65] Benjamini Y, Hochberg Y (1995). Controlling the false discovery rate: a practical and powerful approach to multiple testing. J R Stat Soc Ser B.

[66] Landrigan J-F, Bell T, Crowe M, Clay OJ, Mirman D (2019). Lifting cognition: a meta-analysis of effects of resistance exercise on cognition. Psychol Res.

[67] Smit CAJ, Berenpas F, de Groot S, Stolwijk-Swuste JM, Janssen TWJ (2020). Feasibility of overnight electrical stimulation-induced muscle activation in people with a spinal cord injury. A pilot study. Spinal cord Ser cases..

[68] Wijker BJ, de Groot S, van Dongen JM, van Nassau F, Adriaansen JJE, Achterberg-Warmer WJ (2022). Electrical stimulation to prevent recurring pressure ulcers in individuals with a spinal cord injury compared to usual care: the Spinal Cord Injury PREssure VOLTage (SCI PREVOLT) study protocol. Trials.

